# Challenge in Diagnosis and Treatment of Ectopic Hepatocellular Carcinoma: A Case Report and Literature Review

**DOI:** 10.3389/fsurg.2022.827006

**Published:** 2022-03-31

**Authors:** Qicen Liu, Jingyi Li, Yi Pan, Xiang Zheng, Bin Gao

**Affiliations:** ^1^Department of General Surgery, Affiliated Hangzhou First People's Hospital, Zhejiang University School of Medicine, Hangzhou, China; ^2^Department of Emergency Intensive Care Medicine, The Fifth People's Hospital of Shanghai, Fudan University, Shanghai, China; ^3^Department of Pathology, Huangshan People's Hospital, Huangshan, China; ^4^Department of Chronic Wound Diagnosis and Treatment Center, Affiliated Hangzhou First People's Hospital, Zhejiang University School of Medicine, Hangzhou, China; ^5^Department of Vascular Surgery, Zhongshan Hospital, Fudan University, Shanghai, China

**Keywords:** ectopic hepatocellular carcinoma, ectopic liver, pancreatic tumor, case report, literature review

## Abstract

**Background:**

Findings of ectopic hepatocellular carcinoma (EHCC) have been rarely documented. Complicated clinical features and unpredictable medical prognosis make diagnosis and treatment difficult.

**Case Presentation:**

We reported a 59-year-old male patient who came to the hospital with epigastric discomfort and regurgitation of gastric acid. An enhanced CT scan revealed a 1.8 cm × 1.4 cm mass in the tail of pancreas without any positive finding in the liver. Postoperative MRI scan was performed but did not reveal any evidence of hepatic tumor. The tumor was resected *in toto*. Meanwhile, a 1 cm × 1 cm mass in the body of the stomach was found that was removed simultaneously. Histopathology showed that the pancreatic tumor was ectopic hepatocellular carcinoma (EHCC), and that the gastric nodule was gastrointestinal stromal tumor (GIST). The patient had an uneventful postoperative recovery. He has been living without recurrence for over 7 years since surgery. Owing to our knowledge, this is the second-longest disease-free survival time for EHCC in the literature.

**Conclusion:**

Here, we present a rare case of EHCC in the pancreas, and review the current literature on EHCC. Operation was an effective treatment for patients with curable EHCC. EHCC with metastasis still needs more practice to improve the poor prognosis.

## Introduction

The finding of ectopic liver accidentally happens in abdominal surgery and autopsy. It develops in various locations such as the gallbladder, intra-abdominal ligaments, omentum, peritoneum, retroperitoneum, and thorax (1–3). Nevertheless, lesions have a tendency to develop into hepatoma without mother liver malignancy. The underlying mechanism is unclear probably because of compromised vascular supply or biliary drainage (4).

The diagnosis of EHCC before a pathological result is complicated because of various clinical features. Blood tests and imaging cannot give a significant clue. At the same time, treatment for EHCC does not have a gold standard, and it still needs more practice. In this study, we report a rare case of a combination of ECHH and GIST, which were both successfully treated with tumor resection and 7 years follow-up without recurrence. After the case presentation section, a detailed literature review of EHCC was performed.

## Case Presentation

**Figure 1:**
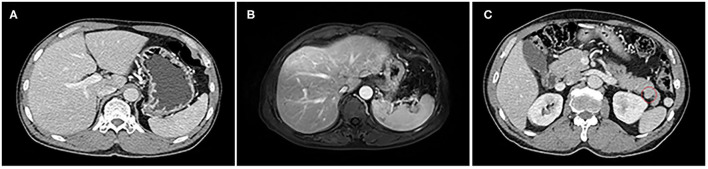
The liver with standard size and shape on CT and MRI; there are no symptoms of liver cirrhosis that can be observed. **(A)** Abdominal CT scan. **(B)** MRI scan. A tiny oval and smooth tumor was shown in the enhanced CT scan **(C)**.

A 59-year-old man visited our hospital with epigastric discomfort and regurgitation of gastric acid in April 2014. There were no remarkable findings in the past medical history. He denied alcohol and drug abuse history. The abdomen was soft and without palpable mass. Tests for hepatitis B virus and hepatitis C virus were negative. Other laboratory examinations were regular, including low levels of CEA, CA19-9, and AFP (AFP: 3.1 ng/ml, CEA: 5.3 ng/ml, and CA199: 12.7 U/ml). No significant indication was observed in hepatic function and routine blood examination. The contrast-enhanced computed tomography (CT) demonstrated a slight oval mass with irregular enhancement, which measured ~1.8 cm × 1.4 cm in the tail of pancreas. Moreover, impression of pancreatic duct dilation or stenosis was not detected by magnetic resonance imaging (MRI). By careful evaluation with the radiologist, there was no noticeable abnormality in the liver shown on CT. The postoperative contrast-enhanced magnetic resonance imaging (MRI) also confirmed the speculation on the hepatic condition (Figure 1).

**Figure 2:**
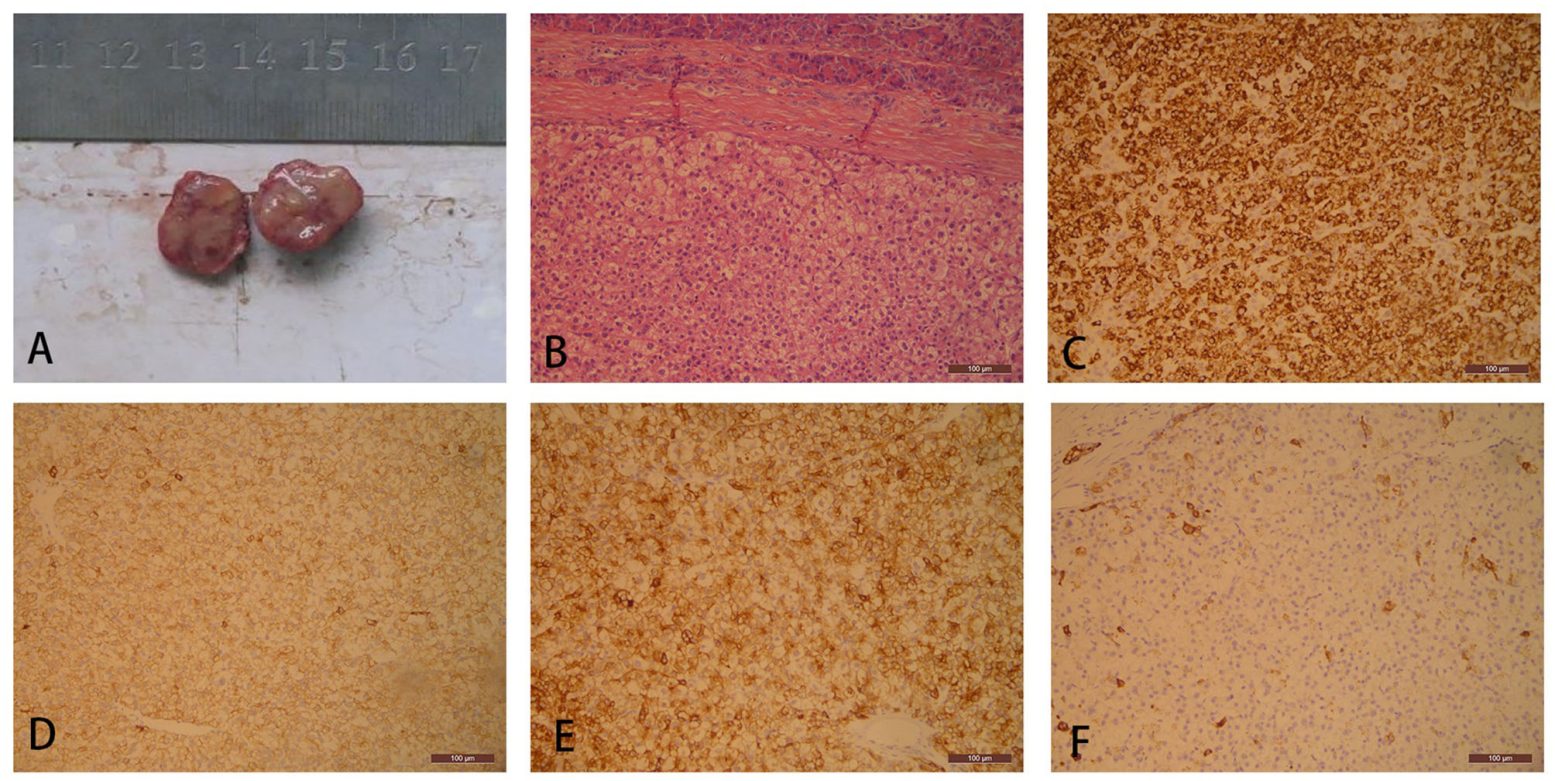
Gross specimen of a pancreatic tumor consists of a well-demarcated and solid firm mass with a yellow appearance **(A)**. Pancreatic tumor cells are separated from normal pancreatic tissue with a prominent fibrous capsule. The tumor cells are polygonal, densely arranged, and abundant eosinophilic granular cytoplasm with partial cellular edema (hematoxylin and eosin, H&E, ×100) **(B)**. Ectopic hepatocellular carcinoma (HCC) demonstrates simultaneous cytoplasm positive for **(C)** HepPar1, **(D)** CK, **(E)** CK8, and **(F)** CK19.

Traditional laparotomy was performed, which revealed nothing but a tumor in the pancreatic space that was mentioned in previous CT. The surface of the tumor was yellow and well-demarcated, and had a focal hemorrhagic appearance (Figure 2). The process of separation of the tumor is smooth without adhesion. There was no sign of invasion to the pancreas or other adjacent organs. Hepatic palpation was regular during the surgery, without cirrhosis and other abnormal findings. No lesion was found in the mother liver. Meanwhile, a lump like a GIST was found in the pylorus. Contrary to the hard texture of the pancreatic tumor, the touch of the stomach tumor is between soft and hard. Both tumors were resected with an adequate margin. Microscopy of the pancreatic tumor showed pleomorphic and prominent nuclei and eosinophilic cytoplasm with a section of pancreatic tissue that separated with capsule (Figure 2). The immunohistochemistry of pancreatic tumor was positive for Hep-Par-1, CK, CK8, and CK19, and negative for AFP, S100, Vim, CgA (chromogranin A), and P53 (Figure 2). The final diagnosis was ectopic HCC arising in the tail of pancreas and GIST in the stomach. Postoperative recovery was well without further chemotherapy therapy. Surveillance of AFP, ultrasound, and CT failed to find a sign of recurrence. Up to now, relapse was not observed 7 years after the operation.

Consent for publication was signed by our patient and approved by the Ethics Committee of the Fifth People's Hospital of Shanghai.

## Literature Review

We searched MEDLINE and EMBASE in February 2022 and the search strategy was “ectopic” and “hepatocellular” and “carcinoma” in title, abstract, and key-words. Included cases must provide confirmed pathological images, follow-up examination, and treatment methods. In the end, 27 cases were available for the criteria (including our case) (3, 5–27). Among these cases, male to female ratio was 2:1. Age ranged from 34 to 81 years (median age 61.6 years). Twelve patients were Caucasian and 15 were Asian. Twenty-five patients were detected with hepatitis. Five of these patients had HBV or HCV infection. AFP level was elevated in 13 of 21 patients who had a serological test (AFP > 20 ng/ml). The overall survival rate of 27 patients at 1 and 3 years was 92.6% and 85.2%. Recurrence appeared in 6 of 24 curable patients. Four patients died in our review. The longest follow-up is Takayasu's patient of 8 years, and our case was the second longest follow-up of 7 years (26). The detail of literature review is listed in Tables 1, 2.

**Table 1:** Literature review of ectopic hepatocellular carcinoma (EHCC).

**Case**	**References**	**Age (year)**	**Sex**	**Country**	**Size of tumor (cm)**	**Location**	**Virus marker**	**Metastasis**	**AFP (ng/mL)**	**Treatment**	**Follow-up**	**Outcome**
1	Wei et al. (18)	71	M	USA	9.1	In right adrenal	Negative	No	Normal	Radical resection	10 months	Recurrence and still alive
2	Ko et al. (10)	73	M	Japan	5.5	Multiple within the peritoneum	Negative	YES	1,164	Diagnostic resection + sorafenib	12 months	Died
3	Adachi et al. (3)	81	F	Japan	7.5	Head of the pancreas	HCV infection	No	30.1	Radical resection	8 months	No recurrence
4	Lee et al. (11)	65	M	South Korea	3.8	Left subphrenic region	Negative	No	N/A	Radical resection	17 months	No recurrence
5	Braun et al. (6)	77	M	Poland	2.5	Tail of the pancreas	Negative	No	Normal	Radical resection	24 months	No recurrence
6	Jin et al. (8)	56	M	China	30	Multiple within abdominal cavity	Negative	Yes	Normal	Radical resection + chemotherapy and TACE	22 months	Recurrence and died
7	Li et al. (12)	44	F	China	5	Adject to pancreas	Negative	No	553.9	Radical resection	17 months	No recurrence
8	Cui et al. (7)	63	M	China	4.6	Thoracic and abdominal cavities	HBV infection	Yes	24,793	Palliative surgery + sorafenib	13 months	Progression free survival (PFS)
9	Soof et al. (17)	69	M	USA	5.7	Pancreatic body/tail	N/A	No	Normal	Radical resection	4 months	No recurrence
10	Aarås et al. (5)	64	F	Norway	3.5	Between stomach and diaphragm	Negative	No	200	Radical resection	48 months	Recurrence and still alive
11	Segura-Sánchez et al. (15)	49	F	Spain	12	In the gallbladder	Negative	No	13,785	Radical resection	36 months	No recurrence
12	Nenekidis et al. (14)	68	M	Greece	7	Pleural cavity and skull	Negative	Yes	elevated	Radical resection	24 months	No recurrence
13	Singh et al. (16)	60	M	India	8	Suprarenal region	HBV infection	No	35	Radical resection	6 months	Recurrence and died
14	Kanzaki et al. (9)	59	F	Japan	2	Below the left diaphragm	Negative	No	2,508	Radical resection	18 months	No recurrence
15	Matsuyama et al. (13)	69	M	Japan	25	Both in the spleen and lung	N/A	Yes	N/A	Chemotherapy	21 months	Died
16	Huang et al. (21)	62	F	China	16	Diaphragm	Negative	No	45,000	Radical resection + chemotherapy	8 months	No recurrence
17	Kubota et al. (23)	56	M	Japan	6.3	In the pancreatic tail	Negative	No	N/A	Radical resection	36 months	No recurrence
18	Cardona et al. (20)	58	M	USA	3.3	Body of the pancreas	Negative	No	Normal	Radical resection	15 months	No recurrence
19	Shigemori et al. (25)	72	M	Japan	14	Jejunum	Negative	No	99,100	Radical resection + TACE	12 months	No recurrence
20	Tsushimi et al. (27)	72	F	Japan	2.5	Bile duct	Negative	No	N/A	Radical resection	12 months	No recurrence
21	Leone et al. (24)	34	F	Italy	10	Beneath the left diaphragm	Negative	No	N/A	Radical resection+ chemotherapy	48 months	Recurrence and still alive
22	Leone et al. (24)	62	M	Italy	9	Left upper abdomen	Negative	No	4,000	Radical resection	48 months	No recurrence
23	Leone et al. (24)	54	F	Italy	9	Within gallbladder	Negative	No	N/A	Radical resection	48 months	No recurrence
24	Kim et al. (22)	43	M	South Korea	10	Beneath the left diaphragm	HBV infection	No	Normal	Radical resection	23 months	Recurrence and still alive
25	Asselah et al. (19)	66	M	France	17	Left chest wall	HCV infection	No	Normal	Radical resection	12 months	No recurrence
26	Takayasu et al. (26)	57	M	Japan	5	Beneath the left diaphragm	Negative	No	2,207	Radical resection	96 months	No recurrence
27	Our case/2021	59	M	China	1.8	Tail of the pancreas	Negative	No	Normal	Radical resection	84 months	No recurrence

*N/A, none of available. Outcome means the time from initial treatment to the ending of follow-up*.

**Table 2:** Summary of literature review information.

**Variables**	**No. of patients (*n* = 27)**
Age, years*	61.6 ± 10.5 (34–81)
**Sex**	
Male	18
Female	9
**Race**	
Caucasian	12
Asian	15
**Hepatitis virus test**	25
**HBV infection**	3
**HCV infection**	2
**Blood test of AFP**	21
**Elevated (>20 ng/mL)**	13
**Normal**	8
Size of tumor, cm*	8.7 ± 6.7(1.8–30)
**Patient with metastasis**	5
Follow-up, months*	26.7 ± 22.4(4–96)
**Overall survival**	
1-year survival rates	92.6%
3-year survival rates	85.2%

*Data are given as mean ± SD (range).

## Discussion

As it is known, ectopic liver tissue and development of cancer from ectopic liver tissue is a rare condition (28). The ectopic liver is regarded as an abnormal tissue, which is similar to liver tissue of morphology and histology, having no linking structure to the mother liver. According to Martinez's report, the incidence of ectopic tissue ranges from 0.24 to 0.47% (29). About 70 patients were detected with an ectopic liver, and 9 of them were diagnosed with EHCC (30). Thus, ectopic liver tissue is considered as having high propensity for hepatocellular carcinoma. This phenomenon is possibly due to pathology difference, which usually appears as abnormality of vascular supply or biliary drainage (4).

What made ectopic liver occur is still unclear. However, the embryology may give us a better understanding of ectopic liver. The original structure of the liver is divided in the caudal part of the foregut during the 4th week of embryonic development. At that time, the primary liver encounters the diaphragm, ventral mesentery, gallbladder, extrahepatic biliary duct, and pancreas (31). According to congenital theory, any variation in hepatic diverticulum development would lead to liver ectopy. Therefore, gallbladder is the most frequent place for finding ectopic liver based on its closest position to the original liver in the embryonic development period. In contrast, transdifferentiation of hepatopancreatic stem cells from the pancreas to the liver is another possible etiology (32). Transformation from pancreatic progenitor cells into hepatocytes has already succeeded *in vitro* (33, 34). It could explain the abnormal phenomenon with multipotent/stem cell theory.

Hepatoid carcinoma is another histological subtype that mimics the morphology and histology of HCC and demonstrates a trabecular, medullary, ductal, glandular, or endocrine component (35). In contrast, EHCC seems to have pure HCC represented without mixed components. For these two subtypes, IHC results of hepatocyte paraffin (HepPar1/HepPar6) and α-fetoprotein (AFP) usually are both positive (36). Gurzu et al. (37) proved that the cytoplasmic expression of VSIG1/TTF1 is the pivotal biomarker of the development of gastric-type HCCs and hepatoid carcinomas. It may be a potential biomarker that could be used for differential diagnosis of these two subtypes. Furthermore, hepatoid carcinoma, regarded as poorly differentiated adenocarcinoma, is commonly related to unfavorable clinical outcomes, while ectopic HCC always shows better prognosis.

In reviewing, the clinical features of EHCC, the most common clinical manifestations were dizziness, poor appetite, nausea, asymptomatic palpable mass, and abdominal pain (12, 13, 22, 25). The early symptom of EHCC is hard to find, since the presentation is often silent until symptoms such as compression, pain, and bleeding occur. It is usually in the advanced stage when patients come to a hospital (8, 10). Our patient visited the hospital because of epigastric discomfort and regurgitation. It was confirmed that atypical symptoms were usual in EHCC, making them barely able to find early. In an early report, majority of patients with EHCC were of Asian race, and in some reports were Caucasians (20). However, in our literature review, data on Caucasians were 12 of 27 cases (44%). The contradictory data might be due to the greater number of worldwide cases we collected. They made the clinical information seem convincing.

Because of complicated clinical manifestations, only a few clues for diagnosis could be given to doctors. Radiography and laboratory examination rarely help in confirming the diagnosis. Proving EHCC is difficult without biopsy or operation. Only 3 of the 27 patients underwent fine needle aspiration (FNA). Two of three cases were diagnosed with EHCC before treatment or operation (3, 14, 16). Therefore, FNA could be an effective detection tool when a tumor cannot be classified. Immunohistochemistry of EHCC was almost positive for hepatocyte or hepatocyte-paraffin-1 (16 of 23 cases in our review). Thus, it is a vital marker for EHCC diagnosis. On the other hand, in 13 of 21 cases, AFP level was elevated (62%, AFP > 20 ng/ml). The data are similar to those on HCC in Fabio's report (54%, AFP > 20 ng/ml) (38). The traditional testing of AFP, which is always elevated in HCC, can also be evidence for suspected EHCC and a follow-up index.

Twenty-six patients underwent operations in our review, and radical resection was performed on 24 patients. Majority of these patients who underwent a radical operation recovered well. At the same time, six of the 24 patients had recurrence after radical resection (16, 18, 22). Obviously, operation is still the first choice for curable patients. Several patients had a recurrence of HCC in the mother liver after operation (16, 22). Although no lesion was found in the mother liver at the time of initial treatment, TACE was an option to prevent recurrence. Metastatic EHCC was reported on 5 patients (7, 8, 10, 13, 14). Only one of them showed no sign of recurrence after treatment (14). This patient was an old male who had tumors in the chest cavity and skull. Only resection of both tumors was performed. Two-year follow-up did not find any sign of recurrence. Although the remaining four patients received chemotherapy, TACE, or Sorafenib for systemic therapy, three died within 2 years. Based on the above information, metastatic EHCC is still a challenge for doctors. On the other hand, a favorable outcome showed the overall survival rate at 1 and 3 years (92.6 and 85.2%) for all the 27 patients. In contrast to a study on 1,492 patients with HCC, the 1- and 3-year overall survival rate of the patients was 96.6 and 88.8% (39). The concordant result suggested that EHCC without metastasis would have a favorable outcome as HCC expressed. Precise information on the prognosis of EHCC is still unclear, and long-term follow-up is required to evaluate various treatments.

## Conclusion

The recommendation for diagnosis of EHCC is biopsy before operation. The AFP of blood test and hepatocyte-paraffin-1 of IHC are valuable for a definitive diagnosis of EHCC. Radical operation is necessary for a curable case. TACE, chemotherapy, and targeted therapy (like Sorafenib) are optional for patients with metastasis or multiple nodules. More cases of EHCC still need to be recorded to evaluate the prognosis and option for different treatments, especially long-time surveillance.

## Data Availability Statement

The original contributions presented in the study are included in the article/supplementary material, further inquiries can be directed to the corresponding author/s.

## Ethics Statement

The studies involving human participants were reviewed and approved by Ethics Committee of The Fifth People's Hospital of Shanghai. The patients/participants provided their written informed consent to participate in this study. Written informed consent was obtained from the individual(s) for the publication of any potentially identifiable images or data included in this article.

## Author Contributions

BG and JL collected the data. QL analyzed the data and wrote the manuscript. BG, XZ, YP, and JL revised the important intellectual content and helped in the final approval of the article. All authors read and approved the final version of the manuscript.

## Conflict of Interest

The authors declare that the research was conducted in the absence of any commercial or financial relationships that could be construed as a potential conflict of interest.

## Publisher's Note

All claims expressed in this article are solely those of the authors and do not necessarily represent those of their affiliated organizations, or those of the publisher, the editors and the reviewers. Any product that may be evaluated in this article, or claim that may be made by its manufacturer, is not guaranteed or endorsed by the publisher.

## Abbreviations

EHCC: ectopic hepatocellular carcinoma
GIST: gastrointestinal stromal tumor
HCC: hepatocellular carcinoma
TACE: transhepatic arterial chemotherapy and embolization
IHC: immunohistochemistry
Hep-Par-1: hepatocyte-paraffin-1
AFP: alpha-fetoprotein
FNA: fine needle aspiration
H&E: hematoxylin and eosin stain.
